# An update on polygalacturonase-inhibiting protein (PGIP), a leucine-rich repeat protein that protects crop plants against pathogens

**DOI:** 10.3389/fpls.2015.00146

**Published:** 2015-03-20

**Authors:** Raviraj M. Kalunke, Silvio Tundo, Manuel Benedetti, Felice Cervone, Giulia De Lorenzo, Renato D'Ovidio

**Affiliations:** ^1^Dipartimento di Scienze e Tecnologie per l'Agricoltura, le Foreste, la Natura e l'Energia, Università della TusciaViterbo, Italy; ^2^Dipartimento di Biologia e Biotecnologie “Charles Darwin”, Sapienza Università di RomaRoma, Italy

**Keywords:** polygalacturonase inhibiting proteins (PGIPs), gene family, transgenic plants, plant protection, fungal pathogens, bacterial pathogens

## Abstract

Polygalacturonase inhibiting proteins (PGIPs) are cell wall proteins that inhibit the pectin-depolymerizing activity of polygalacturonases secreted by microbial pathogens and insects. These ubiquitous inhibitors have a leucine-rich repeat structure that is strongly conserved in monocot and dicot plants. Previous reviews have summarized the importance of PGIP in plant defense and the structural basis of PG-PGIP interaction; here we update the current knowledge about PGIPs with the recent findings on the composition and evolution of *pgip* gene families, with a special emphasis on legume and cereal crops. We also update the information about the inhibition properties of single *pgip* gene products against microbial PGs and the results, including field tests, showing the capacity of PGIP to protect crop plants against fungal, oomycetes and bacterial pathogens.

## Introduction

Successful colonization of plant tissues by microbial pathogens requires the overcoming of the cell wall. To this end, pathogens produce a wide array of plant cell wall degrading enzymes (CWDEs), among which endo-polygalacturonases (PGs; EC 3.2.1.15) are secreted at very early stages of the infection process (ten Have et al., 1998). PGs cleave the α-(1–4) linkages between the D-galacturonic acid residues of homogalacturonan, the main component of pectin, causing cell separation and maceration of the host tissue. To counteract the activity of PGs, plants deploy the cell wall polygalacturonase inhibiting proteins (PGIPs) that inhibit the pectin-depolymerizing activity of PGs. No plant species or mutants totally lacking PGIP activity have been characterized so far. The structure of PGIPs is typically formed by 10 imperfect leucine-rich repeats (LRRs) of 24 residues each, which are organized to form two β-sheets, one of which (sheet B1) occupies the concave inner side of the molecule and contains residues crucial for the interaction with PGs (Di Matteo et al., 2003). In addition to PG inhibition, the interaction between PGs and PGIPs promotes the formation of oligogalacturonides (OGs), which are elicitors of a variety of defense responses (Cervone et al., 1989; Ridley et al., 2001; Ferrari et al., 2013). Since many aspects of the PGIP biology have been already summarized in previous reviews (De Lorenzo et al., 2001; De Lorenzo and Ferrari, 2002; D'Ovidio et al., 2004a; Gomathi and Gnanamanickam, 2004; Shanmugam, 2005; Di Matteo et al., 2006; Federici et al., 2006; Cantu et al., 2008; Misas-Villamil and van der Hoorn, 2008; Protsenko et al., 2008; Reignault et al., 2008; Lagaert et al., 2009), here we present an overview of the recent findings on genome composition and evolution of *pgip* gene families and on the efficacy of PGIP to limit the development of diseases caused by microbial pathogens in crop plants.

## *PGIP* genes and their genomic organization

Early characterization of a polygalacturonase-inhibiting activity was reported in 1970s (Albersheim and Anderson, 1971) and the first *pgip* gene was isolated 20 years later in French bean (Toubart et al., 1992). Since then, several PGIPs and a large number of *pgip* genes have been characterized. Up to now more than 170 complete or partial *pgip* genes from dicot and monocot plants have been deposited in nucleotide databases (e.g., http://www.ncbi.nlm.nih.gov/). Most of these genes have been identified as *pgip* genes on the basis of sequence identity but only a few of them have been shown to encode proteins with PG-inhibitory activity.

**Figure 1:**
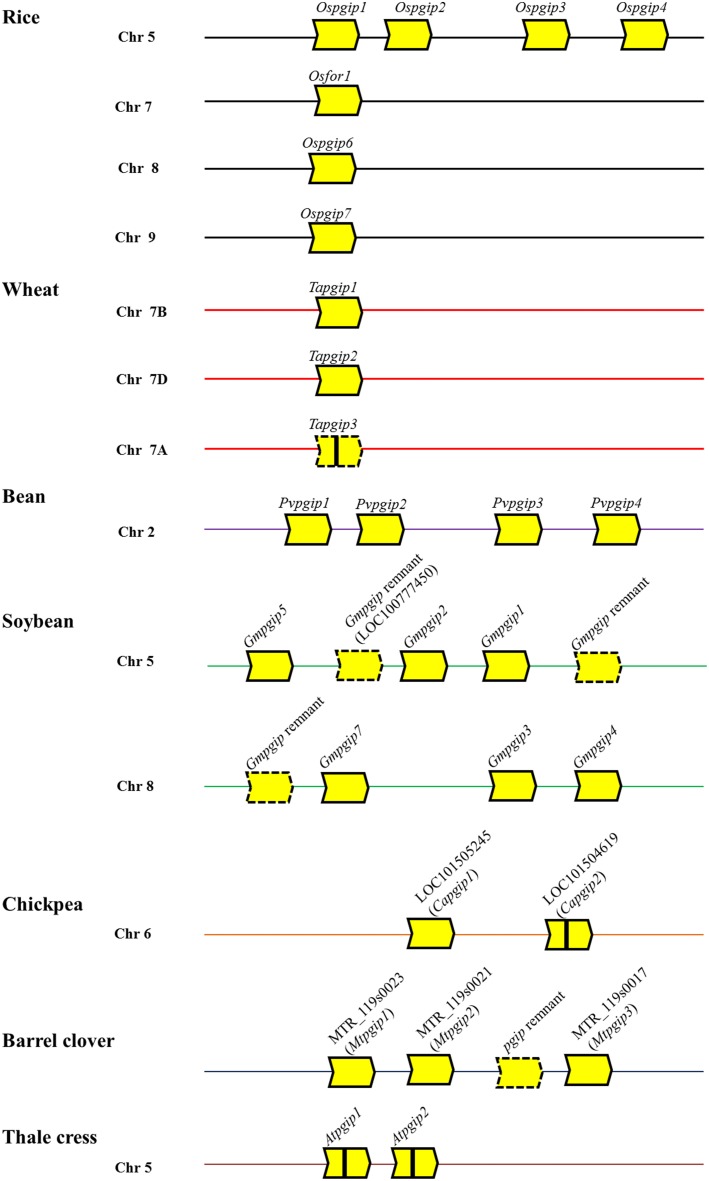
**Schematic representation of the genomic organization *pgip* families in rice, wheat, bean, soybean, chickpea, barrel clover and thale cress**. Each block-arrow with compound-type lines represents a predicted *pgip* gene and a block-arrow with dash type lines represents a predicted pseudo-gene or remnant gene. Vertical line within block-arrow indicates introns (*Capgip2*, *Atpgip1*, and *Atpgip2*) or a Copia retrotransposon (*Tapgip3*). The direction of the arrow indicates ATG to stop codon. The location of *pgip* genes of legume species are based on Kalunke et al. (2014), those of rice and wheat on Janni et al. (2006) and Di Giovanni et al. (2008), and those of thale crest on Ferrari et al. (2003). Chr, chromosome.

Genome analysis has shown that *pgip* genes did not undergo a large expansion and may exist as single genes, as in diploid wheat species (Di Giovanni et al., 2008), or organized into gene families, the members of which are organized in tandem and can vary from two, as in *Arabidopsis thaliana* (Ferrari et al., 2003), to sixteen, as in *Brassica napus* (Hegedus et al., 2008). The majority of *pgip* genes are intronless, however, some of them can contain a short intron as in *Atpgip1* and *Atpgip2* (Ferrari et al., 2003). Moreover, *pgip* genes can be inactivated by transposon elements as in cultivated and wild wheat where the occurrence of *Copia*-retrotransposon and *Vacuna* transposons has been reported (Di Giovanni et al., 2008). Characterized *pgip* loci are shown in Figure 1. Like other families of defense-related genes, *pgip* families show variation in the expression pattern of the different members, some of which are constitutive, others are tissue-specific and, in most cases, up-regulated following stress stimuli (see reviews indicated above; Table 1). At the protein level, members of a *pgip* family show both functional redundancy and sub-functionalization (De Lorenzo et al., 2001; Federici et al., 2006). As suggested previously, these features likely have an adaptive significance for combating more efficiently a broad array of pathogens (Ferrari et al., 2003) or responding more rapidly to diverse environmental stimuli (D'Ovidio et al., 2004b). In support of this view, a recent analysis of the genomic organization and composition of the legume *pgip* families suggested that the forces driving the evolution of the *pgip* genes follow the birth-and-death model (Kalunke et al., 2014), similarly to what proposed for the evolution of NBS-LRR-type *R* genes (Michelmore and Meyers, 1998). This possibility is based on genomic features that include inferred recent duplications, diversification as well as pseudogenization of *pgip* copies, as found in soybean, bean, barrel clover and chickpea (Kalunke et al., 2014). The organization of the *pgip* families therefore supports the view that tandem duplications are frequent in stress-related genes and are beneficial for survival in challenging environments (Oh et al., 2012).

**Table 1:** **Treatments or stress stimuli affecting *pgip* expression in some plant species with a well characterized pgip family**.

***Pgip* family**	**Treatments or stress stimuli**	**References**
Rice	Abscisic acid (ABA), brassinosteroid, gibberellic acid (GA), 3-indole acetic acid (IAA), jasmonic acid (JA), kinetin, naphthalene acetic acid (NAA), salicylic acid (SA); *Rhizoctonia solani* (necrotrophic fungus)	Janni et al., 2006; Lu et al., 2012
Wheat	*Bipolaris sorokiniana* (necrotrophic fungus) and mechanical wounding	Janni et al., 2013
Bean	Oligogalacturonides (OGs); mechanical wounding; *Botrytis cinerea*, *Sclerotinia sclerotiorum* (necrotrophic fungi); *Colletotrichum lindemuthianum* (hemibiotrophic fungus)	Bergmann et al., 1994; Nuss et al., 1996; Devoto et al., 1997; D'Ovidio et al., 2004b; Oliveira et al., 2010; Kalunke et al., 2011
Soybean	Mechanical wounding; *S. sclerotiorum* (necrotrophic fungus)	D'Ovidio et al., 2006; Kalunke et al., 2014
*M. truncatula*	JA, SA, ABA; *Colletotrichum trifolii* (hemibiotrophic fungus)	Song and Nam, 2005
Rapeseed	JA, SA, mechanical wounding; *S. sclerotiorum*	Hegedus et al., 2008
Pepper	SA, Methyl jasmonate (Me-JA), ABA, wounding, cold treatment	Wang et al., 2013
*Arabidopsis*	OGs; JA; *B. cinerea; Stemphylium solani* (necrotrophic fungus); aluminum, low-pH, cold; geminivirus	Ferrari et al., 2003; Ascencio-Ibanez et al., 2008; Sawaki et al., 2009; Di et al., 2012; Kobayashi et al., 2014

## Inhibition activity of PGIPs

A number of papers deals with the inhibition activity of PGIPs purified from several plant tissues. This aspect has been reviewed several years ago (De Lorenzo et al., 2001); here, we present an update of this information (Table 2). Because purified PGIPs may contain a mix of highly similar PGIP isoforms, the activity detected in a tissue may result from the contribution of the activities of different PGIPs expressed in that tissue. An appropriate approach to study the inhibition activity of individual PGIP isoforms is their expression in a heterologous system. However, only a few of the more than 170 *pgip* genes isolated so far from different plant species have been investigated. As reported in Table 3, individual heterologous expression and analysis of all members of a *pgip* family has been performed only for *Arabidopsis* (Ferrari et al., 2003), common bean (D'Ovidio et al., 2004b), soybean (D'Ovidio et al., 2006; Kalunke et al., 2014) and wheat (Janni et al., 2013). PGIPs have been expressed in prokaryotic systems, as a fusion with the maltose-binding protein (MBP) (Jang et al., 2003; Table 3) or using lower temperature for bacterial growth (Chen et al., 2011), in *Pichia pastoris* and in plants by stable transformation or, transiently, by virus-mediated expression (Table 3). In some cases, the proteins were successfully expressed, but did not show any inhibitory activity *in vitro*, as, for example, in the case of some GmPGIPs (D'Ovidio et al., 2006). GmPGIP3, but not GmPGIP1, GmPGIP2, and GmPGIP7 showed inhibitory activity, whereas no expression of GmPGIP5 was obtained (D'Ovidio et al., 2006; Kalunke et al., 2014). Similarly, TaPGIP1 and TaPGIP2, encoded by the two members of the wheat *pgip* family, were successfully expressed but showed no inhibition activity (Janni et al., 2013).

**Table 2:** **Bulk PGIP purified from plants and tested against microbial PGs. These data update those reported in De Lorenzo et al. (2001)**.

**Plant**	**Tissue**	**PGIP preparation**	**Polygalacturonases**		**References**
			**Inhibited**	**Not inhibited**	
Tomato (*Solanum lycopersici* L.)	Stem	Crude extract	*Ralstonia solanacearum*		Schacht et al., 2011
Tobacco (*Nicotiana tabacum* L.)	Nectar		*Botrytis cinerea*		Thornburg et al., 2003
Potato (*Solanum tuberosum* L.)		Gel chromatography	*Aspergillus niger*	*Fusarium solani* (isolate 3122)	Machinandiarena et al., 2001
			*Fusarium moniliforme*^§^		
			*Fusarilm solani* isolate 1402		
Common Bean (*Phaseolus vulgaris* L.)	Leaves	PG-Sepharose chromatography	*Fusarium anthophilum*	*Fusarium verticillioides*	Raiola et al., 2008
			*Fusarium circinatum*	*Fusarium proliferatum* ISPAVEmc 1189	
			*Fusarium subglutinans*.	*Fusarium nygamai*	
			*Fusarium proliferatum* isolate 1152		
			*Fusarium proliferatum* PVS-Fu 64		
			*Fusarium sacchari*		
			*Fusarium fujikuroi*		
			*F. thapsinum*		
			*Fusarium moniliforme*^§^ FC-10	*Fusarium moniliforme*^§^ PD	Sella et al., 2004
Leek (*Allium ampeloprasum* L.)	Basal leaves	Mono-S chromatography		*Fusarium anthophilum*	Raiola et al., 2008
				*Fusarium circinatum*	
				*Fusarium subglutinans*	
				*Fusarium proliferatum*	
				*Fusarium sacchari*	
				*Fusarium fujikuroi*	
				*Fusarium verticillioides*	
				*Fusarium proliferatum* ISPAVEmc 1189	
				*Fusarium nygamai*	
Asparagus (*Asparagus officinalis* L.)	White spear	Mono-S chromatography		*Fusarium anthophilum*	Raiola et al., 2008
				*Fusarium circinatum*	
				*Fusarium subglutinans*.	
				*Fusarium proliferatum*	
				*Fusarium sacchari*	
				*Fusarium fujikuroi*	
				*Fusarium verticillioides*	
				*Fusarium proliferatum* ISPAVEmc 1189	
				*Fusarium nygamai*	
Pepper (*Capsicum annuum L*.)	Fruit	Ion-exchange chromatography	*Colletotrichum gleosporoides*,		Shivashankar et al., 2010
			*Colletotrichum capsici*,		
			*Colletotrichum lindemuthianum*		
			*Sclerotium rolfsi*		
			*Fusarium moniliforme*^§^		
Guava (*Psidium guajava* L.)	Fruit	Purified using a Sephadex G-100	*Aspergillus niger*		Deo and Shastri, 2003
“Oroblanco” grapefruit hybrid (*Citrus grandis* × *C. paradisi* Macf.)	Fruit	Anion exchange chromatography	*Penicillium italicum*		D'hallewin et al., 2004
			*Botrytis cinerea*		
Apple (*Malus domestica* L.)	Fruit		*Colletotrichum acutatum*		Gregori et al., 2008
	Fruit skin	Partial purified	*Botryosphaeria dothidea*	*Glomerella cingulata*	Lee et al., 2006
	Parenchymal tissues	Partial purified	*Monilia fructigena*		Buza et al., 2004
Cantaloupe (*Cucumis melo* L.)	Fruit	Cation exchange chromatography	*Phomopsis cucurbitae*	*Didymella bryoniae*	Fish and Davis, 2004
			*Aspergillus niger*	*Rhizopus* PG	
			*Fusarium solani*	*Fusarium verticillioides*	
Cotton (*Gossypium hirsutum* L.)	Stem	PG-affinity chromatography	*Aspergilus niger*		James and Dubery, 2001
Pear (*Pyrus communis* L.)	Fruit	Partial purified	*Verticillium dahliae*		Ladu et al., 2012; Faize et al., 2003
			*Botrytis cinerea*		
			*Venturia nashicola*		
Pearl millets (*Pennisetum glaucum* (L) R. Br.)	Seedlings	Crude extract	*Aspergilus niger*		Prabhu et al., 2012
Grass pea (*Lathyrus sativus* L.)	Seeds	Gel-filtration chromatography	*Aspergilus niger*		Tamburino et al., 2012
			*Rhizopus spp*		
Orange (*Citrus reticulate* L.)	Fruit	Partial purified	*Diaprepes abbreviatus*		Doostdar et al., 1997
Blue mustard (*Chorispora bungeana*)	Leaves, stem, root	Partial purified	*Aspergillus niger*		Di et al., 2009
			*Stemphylium solani*		
Ginseng (*Panax ginseng* L.)		Crude extract	*Colletotrichum gloeosporioides*		Sathiyaraj et al., 2010
			*Phythium ultimum*		
			*Fusarium oxysporum*		
			*Rhizoctonia solani*		
Bread wheat (*Triticum aestivum* L.)	Leaves	Cation exchange chromatography	*Cochliobolus sativus*	*Aspergillus niger* (EPG I and II)	Kemp et al., 2003
				*Cryphonectria parasitica*	
				*Postia placenta*	
				*Fusarium moniliforme*^§^	
				*Colletotrichum lindemuthianum*	
				*Aspergillus niger* exopolygalacturonase	
				*Ralstonia solanacearum*	
Durum wheat (*Triticum turgidum ssp. dicoccoides*	Leaves	Crude extract	*Fusarium graminearum*	*Fusarium phyllophylum*	Janni et al., 2013
			*Bipolaris sorokiniana*		
			*Stenocarpella maydis*		

§ *Reclassified as Fusarium phyllophilum (Mariotti et al., 2008)*.

**Table 3:** ***Pgip* genes individually expressed in plants or in heterologous systems and tested for inhibition activity against microbial PGs**.

**Species**	**Gene**	**Heterologous systems**	**Origin of purified PG**		**References**
			**Inhibited**	**Not inhibited**	
Common bean (*Phaseolus vulgaris* L.)	PvPGIP1	Transgenic tomato		*Fusarium oxysporum*	Desiderio et al., 1997
				*Botrytis cinerea*	
				*Alternaria solani*	
			*Stenocarpella maydis*		Berger et al., 2000
			*Aspergillus niger*		
	PvPGIP1 PvPGIP2 PvPGIP3 PvPGIP4	PVX/*Nicotiana benthamiana*	*Aspergillus niger*	*Lygus rugulipennis*	D'Ovidio et al., 2006; Frati et al., 2006
			*Fusarium moniliforme*^§^	*Adelphocoris lineolatus*	
			*Stenocarpella maydis*	*Orthops kalmi*	
			*Colletotrichum acutatum*	*Closterotomus norwegicus*	
			*Botrytis cinerea*		
	PvPGIP2	Transgenic wheat	*Bipolaris sorokiniana*	*Claviceps purpurea*	Janni et al., 2008; Volpi et al., 2013
			*F. graminearum*		
		Transgenic *Brassica napus*	*Rhizoctonia solani*		Akhgari et al., 2012
		Transgenic sugarbeet	*Fusarium phyllophilum* FC10		Mohammadzadeh et al., 2012
		PVX/*Nicotiana benthamiana*	*Fusarium phyllophilum* FC-10	*Fusarium phyllophilum* 25305	Mariotti et al., 2008
			*Fusarium phyllophilum* 10241	*Fusarium verticillioides* 62264	
			*Fusarium phyllophilum* 25219	*Fusarium verticillioides* PD	
			*Fusarium phyllophilum* 25218		
Runner bean (*Phaseolus coccineus* L.)	PcPGIP2	PVX/*Nicotiana benthamiana*	*Fusarium moniliforme*^§^		Farina et al., 2009
			*Aspergillus niger*		
			*Colletotrichum lupini*		
			*Botrytis cinerea*		
Tepary bean (*Phaseolus acutifolius* L.)	PaPGIP2	PVX/*Nicotiana benthamiana*	*Fusarium moniliforme*^§^		Farina et al., 2009
			*Aspergillus niger*		
			*Colletotrichum lupini*		
			*Botrytis cinerea*		
Lima bean (*Phaseolus lunatus* L.)	PlPGIP2	PVX/*Nicotiana benthamiana*	*Fusarium moniliforme*^§^		Farina et al., 2009
			*Aspergillus niger*		
			*Colletotrichum lupini*		
			*Botrytis cinerea*		
Soybean (*Glycine max* L.)	GmPGIP1 GmPGIP2	PVX/*Nicotiana benthamiana*		*Sclerotinia sclerotiorum* PGb	D'Ovidio et al., 2006; Frati et al., 2006
				*Sclerotinia sclerotiorum* PGa	
				*Fusarium moniliforme*^§^	
				*Botrytis aclada*	
				*Aspergillus niger*	
				*Botrytis cinerea*	
				*Colletotrichum acutatum*	
				*Fusarium graminearum*	
				*Lygus rugulipennis*	
				*Adelphocoris lineolatus*	
				*Orthops kalmi*	
				*Closterotomus norwegicus*	
	GmPGIP3	PVX/*Nicotiana benthamiana*	*Sclerotinia sclerotiorum* PGb		D'Ovidio et al., 2006; Frati et al., 2006
			*Sclerotinia sclerotiorum* PGa		
			*Fusarium moniliforme*^§^		
			*Botrytis aclada*		
			*Aspergillus niger*		
			*Botrytis cinerea*		
			*Colletotrichum acutatum*		
			*Fusarium graminearum*		
	GmPGIP4	PVX/*Nicotiana benthamiana*		*Sclerotinia sclerotiorum* PGb	D'Ovidio et al., 2006; Frati et al., 2006
				*Sclerotinia sclerotiorum* PGa	
				*Fusarium moniliforme*^§^	
				*Botrytis aclada*	
				*Aspergillus niger*	
				*Botrytis cinerea*	
				*Colletotrichum acutatum*	
				*Fusarium graminearum*	
	GmPGIP7	PVX/*Nicotiana benthamiana*		*Sclerotinia sclerotiorum*	
				*Fusarium graminearum*	Kalunke et al., 2014
				*Colletotrichum acutatum*	
				*Aspergillus niger*	
Pepper (*Capsicum annum* L.)	CaPGIP1 CaPGIP2	*Escherichia coli*	*Alternaria alternata*		Wang et al., 2013
			*Colletotrichum nicotianae*		
Rapeseed (*Brassica napus* L.)	BnPGIP1	*Pichia pastoris*	*Sclerotinia sclerotiorum* PG6		Bashi et al., 2013
Chinese cabbage (*Brassica rapa* L.)	BrPGIP2	Transgenic *Brassica rapa*	*Pectobacterium carotovorum*		Hwang et al., 2010
			*Botryosphaeria dothidea*		
	BrPGIP2	*Escherichia coli*	*Sclerotinia sclerotiorum*		HuangFu et al., 2014
Grapevine (*Vitis vinifera* L.)	VvPGIP1	Transgenic tobacco	*Botrytis cinerea* PGI	*Botrytis cinerea* PG3	Joubert et al., 2006
			*Botrytis cinerea* PG4	*Aspergillus niger* PGII	
			*Botrytis cinerea* PG6		
			*Aspergillus. niger* PGA		
			*Aspergillus niger* PGB		
			*Aspergillus niger* PGI	*Botrytis cinerea* PG2	Joubert et al., 2007
Apple *(Malus domestica* Borkh.)	MdPGIP1	Transgenic tobacco	*Colletotrichum lupini*	*Aspergillus niger*	Oelofse et al., 2006
			*Botryosphaeria obtusa*		
			*Diaporthe ambigua*		
		Transgenic potato	*Verticillium dahliae*		Gazendam et al., 2004
Pear (*Pyrus communis* L.)	PpPGIP	Transgenic grape	*Botrytis cinerea*		Agüero et al., 2005
		Transgenic tomato	*Botrytis cinerea*		Powell et al., 2000
		Transgenic persimmon	*Botrytis cinerea*		Tamura et al., 2004
Raspberry (*Rubus idaeus* L.)	RiPGIP	Transgenic pea	*Stenocarpella maydis*		Richter et al., 2006
			*Colletotrichum lupini*		
Wheat (*Triticum aestivum* L.)	TaPGIP1 TaPGIP2	PVX/*Nicotiana benthamiana*		*Fusarium phyllophylu*	Janni et al., 2013
				*Stenocarpella maydis*	
				*Bipolaris sorokiniana*	
				*Fusarium graminearum*	
Rice (*Oryza sativa* L.)	OsPGIP1	PVX/*Nicotiana benthamiana*	*Sclerotinia sclerotiorum*		Janni et al., 2006
			*Fusarium moniliforme*^§^		
			*Fusarium graminearum*		
			*Aspergillus niger*		
			*Botrytis cinerea*		
	OsFOR1	*Escherichia coli* BL21	*Aspergillus niger* PG		Jang et al., 2003
Pearl millet [*Pennisetum glaucum* (L.) R. Br.]	PglPGIP1	*Escherichia coli SHuffle® T7 Express*	*Aspergillus niger*, AnPGII	*Fusarium moniliforme*, FmPGIII	Prabhu et al., 2014
*Arabidopsis thaliana*	AtPGIP1 AtPGIP2	Transgenic *Arabidopsis*	*Colletotrichum gloeosporioides*	*Aspergillus niger*	Frati et al., 2006; Ferrari et al., 2012, 2003
			*Stenocarpella maydis*	*Fusarium moniliforme*^§^	
			*Botrytis cinerea*	*Lygus rugulipennis*	
			*Fusarium graminearum*	*Adelphocoris lineolatus*	
				*Orthops kalmi*	
				*Closterotomus norwegicus*	

§ *Reclassified as Fusarium phyllophilum FC10 (Mariotti et al., 2008)*.

The absence of inhibition activity *in vitro* may also reflect the possibility that some PGIPs are active only in the *in planta* environment, as suggested by Joubert et al. (2006) in the case of the *Botrytis cinerea* BcPG2 and VvPGIP1 from grapevine (*Vitis vinifera L.)*. These proteins do not interact *in vitro*, although VvPGIP1 reduces symptoms caused by BcPG2 upon co-infiltration in leaves. The number and sources of PGs tested is also limited; only a few studies have been carried out against PGs of bacteria and insects (Doostdar et al., 1997; D'Ovidio et al., 2004b; Frati et al., 2006; Hwang et al., 2010; Schacht et al., 2011; Kirsch et al., 2012). The limitations of data prevents to draw conclusions about correlations between PGIPs of specific plant families and specific pathogens. Notably, PG produced by a highly detrimental pathogen, *Fusarium verticillioides*, is not inhibited by any known PGIP (see Table 2). This PG has been a target of an unsuccessful attempt to render PvPGIP2 an efficient inhibitor against this PG (see below, Benedetti et al., 2011a).

The utilization of *pgip* genes for crop protection relies on the identification of inhibitors with broad specificities against the many PGs produced by phytopathogens and/or the construction of novel PGIPs with stronger and broader inhibitor activity. Many more PGIPs than those reported in Tables 2, 3 exist in nature and are likely to have different specificities against microbial PGs, considering that single amino acid changes are able to change specificity of the inhibitors (Leckie et al., 1999). Searching for PGIPs with novel specificities may allow to count on a much larger reservoir of possible genes for crop protection. A direct and simple strategy to isolate PGIPs with recognition capability against a given PG may be based on affinity chromathography methods, similar to that originally used to purify PGIP from *P. vulgaris* (Cervone et al., 1987), and mass spectrometry. Attempts to drive *in vitro* evolution of PGIPs to generate proteins with improved inhibition properties have not been successful yet (Benedetti et al., 2011a).

The occurrence of PG-inhibiting activity in crude leaf protein extracts of tetraploid wild wheat (*T. dicoccoides*) possessing non functional *pgip* genes (Di Giovanni et al., 2008) suggested the existance of *pgip* genes with a sequence divergent from the classical one. This possibility, which deserves further investigation, is also supported by the finding that the wheat tissue contains PG-inhibiting proteins with N-terminal sequences (Lin and Li, 2002; Kemp et al., 2003) different from TaPGIP1 and TaPGIP2 (Janni et al., 2013) and from the *pgip* sequences reported so far (http://www.ncbi.nlm.nih.gov/nucleotide/). Recently, a wheat gene with some sequence similarity to *pgip* genes has been reported and was shown to be involved in the defense response against *Fusarium graminearum* (Hou et al., 2014).

## Structural studies on the PG-PGIP interaction

Thus, the possibility of engineering new forms of PGIPs depends on the detailed structural knowledge of the PG-PGIP interaction. Several structural studies have been performed (Mattei et al., 2001; King et al., 2002; Benedetti et al., 2011b, 2013; Gutierrez-Sanchez et al., 2012), but a high resolution 3D-structure of the PG-PGIP complex is still missing. The enzyme-inhibitor combinations that have been more extensively investigated, are those that PGIP2 from *Phaseolus vulgaris* (PvPGIP2) forms with PG from *A. niger* (AnPGII), *F. phyllophilum* (FpPG) and *C. lupini* (ClPG). Site-directed mutagenesis has shown that the residues involved in the interaction are located in the concave surface of the inhibitor (Leckie et al., 1999; Federici et al., 2001; Spinelli et al., 2009; Benedetti et al., 2011b, 2013). Computational methods such as the Codon Substitution Model in combination with the Desolvation Energy Calculation and the Repeat Conservation Mapping (RCM; Helft et al., 2011) have pinpointed several residues of PvPGIP2 responsible for the PG-inhibiting activity (Casasoli et al., 2009).

On the other hand, residues of PG that are critical for the interaction with PGIP have been also studied. FvPG is 92.5% identical to FpPG, but is inhibited by neither PvPGIP2 nor other known PGIPs. By both loss- and gain-of-function site-directed mutations, a single amino acid at position 274 of both FvPG and FpPG was demonstrated to act as a switch for recognition by PvPGIP2 (Raiola et al., 2008; Benedetti et al., 2013). Unfortunately, the lack of high-resolution structural information on the PG-PGIP complex does not allow to precisely identify the contacting residue in PGIP. Moreover, both PGs and PGIPs are glycosylated proteins (Caprari et al., 1993; Lim et al., 2009); however, whether glycosylation plays a role in the PGIP-PG interaction requires further investigation. For example, glycosylation in pearl millet PGIP was found to affect pH and temperature stability of the protein but not its capability of inhibiting AnPGII (Prabhu et al., 2015).

A single PGIP may display different mechanisms of PG inhibition (competitive, non competitive and mixed) suggesting that the protein is highly versatile in recognizing different epitopes of various PGs (Federici et al., 2001; King et al., 2002; Sicilia et al., 2005; Bonivento et al., 2008). Consequently, many 3D-models based on docking predictions have been proposed so far (Sicilia et al., 2005; Maulik et al., 2009; Prabhu et al., 2014). Techniques such as the mass amide exchange mass spectrometry in the case of AnPGII and FpPG and the Small Angle X-ray Scattering (SAXS) in the case of FpPG and ClPG have produced models that, in some cases, are discordant. For example, while the mass amide exchange mass spectrometry predicts that the area of FpPG in contact with PvPGIP2 is located at the N-terminus and predominantly on the underside of the enzyme beta-barrel structures (Gutierrez-Sanchez et al., 2012), the SAXS analysis indicates that the protein region in contact with PvPGIP2 is located at the C-terminus of the enzyme and includes the loops surrounding the active site cleft. A site-directed mutagenesis analysis has been used to validate this second view (Benedetti et al., 2013). In general, low resolution techniques such as SAXS analysis or mass amide exchange mass spectrometry require validation by site-directed mutagenesis to locate the contacting residues in a protein complex.

The X-ray crystallography, successfully used to solve several high-resolution structures of PGs (van Santen et al., 1999; Federici et al., 2001; Bonivento et al., 2008) and that of PvPGIP2 (Di Matteo et al., 2003), was so far unsuccessful in the case of the PG-PGIP complex. This is probably due to the intrinsic instability of the PG-PGIP interaction, which only occurs, under apoplastic conditions of pH and ionic strength, through the contact of only a few, sometimes only one, residues (Leckie et al., 1999). The use of a cross-linker for stabilizing the PG-PGIP complex coupled to techniques that allow the protein analysis directly in solution, such as SAXS and NMR spectroscopy (Wand and Englander, 1996; Nietlispach et al., 2004), may be a valid alternative in order to obtain a detailed map of the contacting residues but this requires a subsequent validation by site-directed mutagenesis.

## *PGIPs* engineered in dicot crops

The important role of PGIP in plant defense has been demonstrated by overexpressing *pgip* genes in several plant species. In these experiments, the source of the used genes was either the same plant species utilized for transformation or a different one (Table 4). The transformation of the model plant *A. thaliana* has been particularly useful to highlight the potentiality of several *pgip* genes, namely the endogenous *Atpgip1* and *Atpgip2*, the bean *Pvpgip2* and the rapeseed (*Brassica napus*) *Bnpgip1* or *Bnpgip2*. Arabidopsis plants overexpressing *Atpgip1* or *Atpgip2* showed a significant reduction of disease symptoms caused by *B. cinerea* (Ferrari et al., 2003) and were less susceptible against the hemibiotrophic fungal pathogen *F. graminearum* (Ferrari et al., 2012), the major causal agent of Fusarium head blight (FHB). Conversely, silencing of their expression using an antisense *Atpgip*, led to enhanced susceptibility (Ferrari et al., 2006). Arabidopsis plants expressing *Pvpgip2*, encoding an efficient inhibitor of the *B. cinerea* PG (ten Have et al., 1998), showed reduction of disease symptoms caused by *B. cinerea* and those expressing the rapeseed genes *Bnpgip1* and *Bnpgip2* delayed the symptoms caused by *S. sclerotiorum* (Bashi et al., 2013).

**Table 4:** **List of transgenic crops produced using the gene coding for PGIP and their response to fungal, oomycetes or bacterial phytopathogens**.

**Transgenic crops**	**PGIP gene^c^**	**Tested against fungal, oomycetes or bacterial phytopathogens**	**References**
Tomato^a^ (*Solanum lycopersicum* L.)	PcPGIP	Botrytis cinerea^*^	Powell et al., 2000, 1994
	PvPGIP1	*Fusarium oxysporum* f.sp. *lycopersici*^†^	Desiderio et al., 1997
		*Botrytis cinerea*^†^	
		*Alternaria solani*^†^	
Tobacco^a^ (*Nicotiana tabacum* L.)	PvPGIP2	Botrytis cinerea^*^	Manfredini et al., 2005
		Rhizoctonia solani^*^	Borras-Hidalgo et al., 2012
		Phytophthora parasitica^*^	
		Peronospora hyoscyami^*^	
	CaPGIP1	Alternaria alternata^*^	Wang et al., 2013
		Colletotrichum nicotianae^*^	
	VvPGIP1	Botrytis cinerea^*^	Joubert et al., 2006
	BrPGIP2	Pectobacterium carotovorum^*^	Hwang et al., 2010
Potato^a^ (*Solanum tuberosum* L.)	MdPGIP1 StPGIP	*Verticillium dahliae*^†^ Verticillium dahliae^*^	Gazendam et al., 2004; Guo et al., 2014
*Brassica rapa*^a^	BrPGIP2	Pectobacterium carotovorum^*^	Hwang et al., 2010
Rapeseed^a^ (*Brassica napus* L.)	BnPGIP2	Sclerotinia sclerotiorum^*^	HuangFu et al., 2014
Pea^a^ (*Pisum sativum* L.)	RiPGIP	*Glomus intraradices*^Ψ^	Hassan et al., 2012
Grapevine^a^ (*Vitis vinifera* L.) Rice^a^ (*Oriza sativa* L.)	PcPGIP OsPGIP1	Botrytis cinerea^*^	Agüero et al., 2005; Wang et al., 2014b
		Xylella fastidiosa^*^ Rhizoctonia solani	
Wheat^b^ (*Triticum aestivum* L.*, Triticum durum* Desf.)	PvPGIP2 GmPGIP3	Bipolaris sorokiniana^*^	Janni et al., 2008
		Fusarium graminearum^*^	Ferrari et al., 2012
		*Claviceps purpurea*^†^ Bipolaris sorokiniana^*^ Gaeumannomyces graminis var. tritici^*^	Volpi et al., 2013; Wang et al., 2014a
*Arabidopsis thaliana* L.^a^	PvPGIP2	Botrytis cinerea^*^	Manfredini et al., 2005
	AtPGIP1 AtPGIP2	Fusarium graminearum^*^	Ferrari et al., 2012
	BnPGIP1 BnPGIP2	Sclerotinia sclerotiorum^*^	Bashi et al., 2013

a *The transgenic gene was under control of CaMV 35S promoter*.

b *The transgenic gene was under control of Ubiquitin promoter*.

c *Pc, Pyrus communis; Pv, Phaseolus vulgaris; Ca, Capsicum annum; Vv, Vitis vinifera; Br, Brassica rapa; Md, Malus domestica; St, Solanum torvum; Ri, Rubus idaeus; Ac, Actinidia deliciosa; At, Arabidopsis thaliana; Bn, Brassica napa*.

* *Showed enhanced resistance*.

† *No evidence of enhanced resistance*.

Ψ *No effect on mycorrhization*.

The protective potential of *pgip* genes has also been demonstrated in transgenic crops. The first transgenic crop plant obtained by using a *pgip* gene and tested against pathogenic microorganisms were tomatos expressing PvPGIP1 from *P. vulgaris*. These plants, however, did not show any increased resistance against *Fusarium oxysporum* f. sp. *lycopersici*, *B. cinerea*, and *Alternaria solani*. The negative result was due to the inability of PvPGIP1 to inhibit the PGs secreted by these fungi, as shown by *in vitro* inhibition assays and led to discovery of other forms of PGIPs and eventually to the existence of a complex PGIP family in French bean (Desiderio et al., 1997). A few years later, transgenic tomato plants expressing a pear (*Pyrus communis* L.) PGIP (PcPGIP) capable of inhibiting the PGs secreted by *B. cinerea*, showed a reduction of disease lesions caused by this fungus both on ripening fruit (15% reduction) and leaves (about 25% reduction). The initial establishment of infection was not affected in the transgenic plants but the later colonization of the host tissue was significantly reduced (Powell et al., 2000).

Tobacco has been the most used crop plant for testing the effect of PGIP expression on resistance to pathogens. Constitutive and high-level expression of *Pvpgip2* (from *P. vulgaris*), *Vvpgip1* (from *V. vinifera*), *Capgip1* [from pepper (*Capsicum annum*)] and *Brpgip2* (from *B. rapa*) have been obtained in transgenic tobacco. Plants expressing PvPGIP2 showed about 35% reduction of symptoms caused by *B. cinerea* (Manfredini et al., 2005) and, more recently, were shown to display reduced disease symptoms against *Rhizoctonia solani* and two oomycete pathogens, *Phytophthora parasitica* var. *nicotianae* and the blue mold-causing agent *Peronospora hyoscyami* f. sp. *tabacina* (Borras-Hidalgo et al., 2012). Notably, the experiments against *P. hyoscyami* f.sp. *tabacina* were performed in the field during seasonal conditions that favor the pathogen spreading. In agreement with what observed under controlled conditions, resistance of transgenic plants was comparable to that exhibited by *Nicotiana* species (*N. rustica*, *N. debneyi* and *N. megalosiphon*) that are highly resistant to blue mold disease. These transgenic plants expressing PvPGIP2 represented the first example of PGIP-expressing plants subjected to field trails. Recently, transgenic rice expressing OsPGIP1 showed also improved resistance against *Rhizoctonia solani* in field experiments (Wang et al., 2014b).

Transgenic tobacco plants expressing the grapevine *pgip* gene *Vvpgip1* (Joubert et al., 2006) also showed a reduced (from 47 to 69%) disease susceptibility to *B. cinerea* infection. As for plants expressing PvPGIP2, the resistance phenotype correlated with the accumulation of VvPGIP1 as well as with its capability of inhibiting the activity of PG secreted by *B. cinerea*, namely BcPG1, BcPG3, and BcPG6. Several observations, however, suggest that PGIP may improve resistance by mechanisms other than classical PGIP-PG inhibition. For example, non-infected transgenic tobacco plants expressing *Vvpgip1* show modified expression patterns of genes involved in various metabolic pathways (Alexandersson et al., 2011) and an altered cell wall structure (Nguema-Ona et al., 2013). In these plants, lignin accumulation and arabinoxyloglucan-cellulose re-organization leads to a general strengthening/reinforcing of the cell wall that may contribute to an improved resistance against *B. cinerea*.

A reduction of disease symptoms (about 50%) caused by *Alternaria alternata* and *Colletotrichum nicotianae* was also observed in transgenic tobacco lines expressing the pepper CaPGIP1 and, once again, resistance correlated with the inhibition capacity of purified CaPGIP1 against PG activity of both fungal pathogens (Wang et al., 2013).

Within the Solanaceae family, transgenic potato (*Solanum tuberosum*) plants expressing the gene *StPGIP1* from *S. torvum* showed a 50% reduction of wilt disease symptoms caused by *Verticillium dahliae* and a normal plant growth (Guo et al., 2014). Transgenic potato plants overexpressing the apple *pgip1* gene showed protection against the same fungal pathogen but displayed an extended juvenile phase (Gazendam et al., 2004).

Transgenic grapevine (*V. vinifera*) plants constitutively expressing the pear PcPGIP gene represent an interesting example of the potential of PGIP for protection against pathogens other than fungi and oomycetes. These plants show a delayed development of the Pierce's disease (PD) caused by bacterial pathogen *Xylella fastidiosa* (Agüero et al., 2005). Not only leaf scorching and *Xylella* titre were reduced but also plants showed a better re-growth after pruning compared to infected untransformed controls. Moreover, an inverse dose-effect relationship was shown between development of PD and levels of PcPGIP activity in the tissues. The improved resistance of the grapevine plants expressing PcPGIP against a bacterial pathogen was unexpected, because until then the PGIP inhibition activity was thought to be limited to fungal and insect PGs (Cervone et al., 1990; Johnston et al., 1993; D'Ovidio et al., 2004b). It was later shown that pear PcPGIP inhibits the PG encoded by *X. fastidiosa* and that PG activity is a virulence factor of this pathogen (Roper et al., 2007; Pérez-Donoso et al., 2010). The observation that PcPGIP is present in xylem exudates of non-transgenic scions grafted on transgenic rootstocks expressing PcPGIP suggests that grafting of non transgenic varieties on transgenic rootstocks represents, in this case, a useful agronomical practice for plant protection (Agüero et al., 2005).

The results obtained with *X. fastidiosa* prompted further investigations on the capability of PGIP of controlling bacterial diseases (summarized in Table 4). Transgenic tobacco plants expressing *B. rapa* BrPGIP2 were resistant against *Pectobacterium carotovorum*, the causal agent of the soft rot disease, with a strong reduction (66–88%) of the symptoms as compared to wild-type plants (Hwang et al., 2010). The resistance correlated with the inhibitory activity against *P. carotovorum* PG activity found in the total protein extracts of the transgenic plants (Hwang et al., 2010). Also chinese cabbage (*B. rapa* ssp. *pekinensis*) plants overexpressing BrPGIP2 showed higher resistance against *P. carotovorum* and produced normal looking pods-like structures with no viable seeds. Combination of crossing with non-transgenic plants did not restore fertility of the transgenic plants, suggesting that mechanisms such as ploidy changes occurring during the tissue culture stage or changes in cell-wall architecture of sexual organs are responsible for the abnormality (Hwang et al., 2010).

No phenotypic abnormalities were, instead, found in transgenic tobacco plants expressing BrPGIP2 (Hwang et al., 2010), nor in rapeseed plants overexpressing the *B. napus Bnpgip2*. The latter plants displayed a significant reduction of rot caused by the necrotrophic fungal pathogen *S. sclerotiorum* (HuangFu et al., 2014).

Additional PGIP-transgenic crops include pea (*Pisum sativum* L.), transformed with *Ripgip* from raspberry (*Rubus idaeus* L.) (Richter et al., 2006), persimmon (*Diospyros kaki* L.) and apple (*Malus domestica* Borkh.) transformed with pear PcPGIP (Szankowski et al., 2003; Tamura et al., 2004), sugarbeet (*Beta vulgaris* L.) transformed with bean *Pvpgip2* (Mohammadzadeh et al., 2012), chickpea transformed with either *Ripgip* or a *pgip* gene from kiwi fruit (Senthil et al., 2004), tobacco transformed with PpPGIP gene from *Pyrus pyrifolia* Nakai (Liu et al., 2013) and maize (*Zea mays* L.) transformed with bean *Pvpgip1* (O'Kennedy et al., 2001). The response of these plants to pathogens has not been reported yet. Transgenic pea plants expressing RiPGIP were instead evaluated for their response to beneficial microorganisms. *Glomus intraradices*, an arbuscular mycorrhizal fungus, colonized roots of transgenic plants at an extend comparable to that observed in control non transgenic plants, indicating that the expression of RiPGIP does not affect mycorrhization (Hassan et al., 2012).

## *PGIPs* engineered in monocot crops

Although the low pectin content of cereal species like wheat and rice indicates that this cell wall component may have a marginal role during infection, results show that the expression of PGIP in transgenic plants limits some diseases caused by fungal pathogens (Janni et al., 2008; Ferrari et al., 2012; Wang et al., 2014a,b). In our labs, the bean *Pvpgip2* gene was used under the constitutive promoter of the maize unbiquitin gene (*Ubi-1*) to transform both durum and bread wheat by particle bombardment. PvPGIP2 was correctly targeted to the apoplast and the transgenic plants did not show any major morphological and growth defects. Transgenic wheat showed a significant reduction (46–50%) of foliar spot blotch symptoms caused by the hemibiotrophic fungal pathogen *Bipolaris sorokiniana* and improved resistance (25–30%) against the hemibiotrophic fungal pathogen *F. graminearum* (Ferrari et al., 2012), the major causal agent of FHB in wheat. A reduced degradability of the transgenic tissue by PG treatments correlated with the capacity of PvPGIP2 to inhibit PG activity of *B. sorokiniana* and less strongly PG of *F. graminearum* (Janni et al., 2008; Ferrari et al., 2012). An interesting aspect of the wheat plants expressing PvPGIP2 is that, under moderate infection with *F. graminearum*, the reduced FHB symptoms are concomitant with a greater amount of total starch in the grains as compared to control plants (D'Ovidio et al., 2012). On the other hand, wheat plants expressing PvPGIP2 were susceptible to the biotrophic fungal pathogen *Claviceps purpurea*, the causal agent of ergot disease probably because PvPGIP2 is not able to inhibit the activity of *C. purpurea* CpPG1 and CpPG2 (Volpi et al., 2013). Recently, transgenic wheat expressing the soybean GmPGIP3 was shown to be resistant to both take-all and common root rot diseases caused by the fungal pathogen *Gaeumannomyces graminis* var. *tritici* and *B. sorokiniana*, respectively; symptoms were reduced of about 47–83% and 42–60%, respectively (Wang et al., 2014a). Similarly, the expression of OsPGIP1 in transgenic rice enhanced resistence against *Rhizoctonia solani* in field tests and resistance was related with the expression levels of OsPGIP1 (Wang et al., 2014b).

## Concluding remarks and future challenges

The results reported in this review clearly indicate that PGIP is useful to improve resistance in different crop species. High-level expression of PGIP does not prevent infection but limits significantly the colonization of the host tissue with a consequent positive impact on crop yield and product quality. The efficacy of PGIP to control diseases has been demonstrated against fungi, oomycetes and bacteria and is equally efficient against necrorophic and hemibiotrophic pathogens. The experiments performed with biotrophs do not allow to draw any clear conclusion since the only fungal biotrophic pathogen analyzed, *C. purpures*, produced PG activity that was not inhibited by the PGIP expressed in the transgenic plants (Volpi et al., 2013). The identification and development of PGIPs with stronger and broader inhibitory capacities may be useful to utilize these proteins in crop protection. Germplasm analysis to identify novel PGIPs is still limited (Farina et al., 2009) and the initial attempts to drive *in vitro* evolution of PGIP to generate proteins with improved inhibition properties have not been particularly successful (Benedetti et al., 2011a). Structural studies should be implemented in order to obtain a detailed map of the contacts between various PGs and PGIPs. This is necessary not only for constructing novel inhibitors with stronger activities but also for future programs of genome editing in which the existing genes of a plant species may be ameliorated to better adapt to new virulent strains of microorganisms evolving in nature.

**Figure 2:**
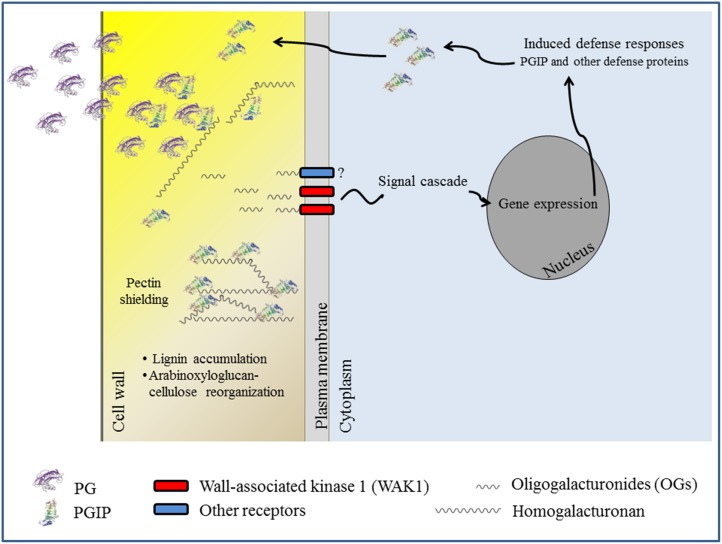
**A model for the role of PGIP in the defense response against pathogens**. Delay of symptoms is related to the inhibitory activity of PGIP toward PGs secreted by the pathogens and likely to the accumulation of oligogalacturonide (OG) elicitors, which are recognized by WAK1 and likely other receptors not yet characterized. Cell wall modification and pectin shielding could also play a role. Signaling cascades activated by OGs are described in Ferrari et al. (2013).

The available results support the notion that inhibition of the microbial PG by PGIP is a prerequisite of the inhibitors to confer resistance to transgenic plants against microbes. The delay of symptoms is often related to the capacity of PGIP to inhibit the PG activity secreted by the pathogens and, consequently, to reduce both tissue maceration and favor the release of OGs, as summarized in Figure 2. However, this aspect of the PGIP's biology needs further investigation. In some cases PGIP has been reported to confer resistance without any evidence of PG-inhibition *in vitro* (Joubert et al., 2006). Moreover, some evidence suggests that the capability of reducing tissue maceration is associated with the property of PGIP to bind pectin, likely shielding this component of the cell wall from PG activity (Spadoni et al., 2006). In this regard the observation that transgenic plants expressing PGIPs exhibit an altered gene expression and cell wall composition is also intriguing. It is not yet clear the mechanism that links the ectopic expression of PGIP to alteration of gene expression and whether this contributes to disease resistance (Alexandersson et al., 2011; Nguema-Ona et al., 2013).

An important but very little explored aspect of the PGIP biology is its possible role in processes of growth and development. Although plants overexpressing PGIPs do not show obvious morphological alterations, indeed several reports point to PGIP as a player in development. PGIP are induced, not only by phosphate deficiency, but also by auxin treatment and in mutants defective in SIZ1, a SUMO (small ubiquitin-related modifier) E3 ligase that is involved in several stress responses, including Pi starvation, and flowering (Sato and Miura, 2011). Suppression of PGIPs under the control ABA insensitive 5 (ABI5) transcription factor accompanies promotion of seed germination by the peroxisomal ABC transporter PED3 (Kanai et al., 2010). Upregulation of *PGIP2* correlates with the acquisition of competence to form green callus in an auxin-rich callus induction medium (Che et al., 2007) and occurs in Arabidopsis tissue culture lines in which the expression of the peroxidases PRX33 and PRX34 is knocked down by antisense expression (O'Brien et al., 2012), whereas PGIP1 was identified in a proteomic study performed on Arabidopsis etiolated hypocotyls used as a model of cells undergoing elongation followed by growth arrest within a short time (Irshad et al., 2008). Finally, both PGIP1 and PGIP2 are associated with cell wall stabilization at low pH under the control of the zinc-finger protein STOP1 (Sensitive to Proton Rhizotoxicity 1) and STOP2 (Kobayashi et al., 2014). A role of PGIP not only in defense but also in growth and development implies that the inhibitor may affect one or more of the many endogenous PGs expressed by plants. This is also an unexplored aspect of the PGIP biology and, at the moment, only one very old evidence is available showing that PGIP may have a plant-derived PG partner (Cervone et al., 1990).

### Conflict of interest statement

The authors declare that the research was conducted in the absence of any commercial or financial relationships that could be construed as a potential conflict of interest.
